# Relapse and Outcome of Lupus Nephritis After Renal Transplantation in the Modern Immunosuppressive Era

**DOI:** 10.7759/cureus.20863

**Published:** 2022-01-01

**Authors:** Debendra Pattanaik, Joseph Green, Manish Talwar, Miklos Molnar

**Affiliations:** 1 Rheumatology, The University of Tennessee Health Science Center, Memphis, USA; 2 Rheumatology, Allina Health, Minneapolis, USA; 3 Surgery, University of Tennessee Health Sciences Center, Memphis, USA; 4 Nephrology and Hypertension, University of Utah Health Sciences Center, Salt Lake City, USA

**Keywords:** immunosuppressive, outcome, relapse, lupus nephritis, kidney transplantation, • kidney transplantation • rheumatoid arthritis • anca- associated vasculitis • living kidney donor • esrd

## Abstract

Background

Recurrence of lupus nephritis in the graft is a concern in lupus patients with end-stage renal disease undergoing renal transplantation. The recurrence of lupus nephritis has been variable among different studies depending on the patient characteristics, immunosuppressive regimen, and indications of renal biopsy. Therefore, we investigated the recurrence of lupus nephritis among our patients to see if the new post-transplant regimen has impacted the recurrence.

Methods

We collected data on all recipients with end-stage renal disease secondary to lupus nephritis, who received renal transplants between 2006-2017 in our center. Patient demographics, transplant, and dialysis-related information have been recorded including kidney biopsy, graft loss, and survival were recorded. An association between recurrent lupus nephritis with survival and/or graft loss was examined using survival models.

Results

The overall mean±SD age at baseline was 42±13 years; 89% were female; 89% were African American; the previous time on dialysis was a median of 4 years (IQR: 2-8 years), 81% received hemodialysis and 31% received living donor transplantation in the cohort. Our patients received the standard immunosuppressive regimen consisting of prednisone, tacrolimus, and mycophenolate mofetil. Four (10.5%) of the 38 patients had biopsy-proven lupus nephritis recurrence. A total of 10 patients (26%) had graft loss or died during the median follow-up time of 1,230 days (IQR: 460-2,227 days). Recurrence of lupus nephritis showed a trend for increased risk of graft loss or patient death (Hazard Ratio: 3.14, 95%Confidence Interval: 0.65-15.24) compared to the recipient without recurrence in our unadjusted proportional Cox regression model.

Conclusion

The recurrence rate of lupus nephritis in our patient population is much lower compared to past studies from different immunosuppressive eras. Patients with recurrent lupus nephritis showed an increased risk of graft loss or death.

## Introduction

Systemic lupus erythematosus (SLE) is a multisystem autoimmune disease characterized by autoantibody formation with heterogeneous clinical manifestations [1]. Approximately 50-60% of patients with SLE develop lupus nephritis (LN) within the first ten years of diagnosis [2]. The five-year risk for developing end-stage renal disease (ESRD) is 10-70% among lupus nephritis patients [3]. Renal transplantation is the treatment of choice for patients with lupus nephritis who develop ESRD [4]. Renal transplantation is superior to dialysis in improving quality of life, survival, and complications [4-7].

The recurrence of LN in the renal allograft has been a concern over the years [5, 8, 9]. Relapse of LN in the allograft increases the risk of graft failure, but the occurrence of graft loss is rare [4]. The recurrence rate in the allograft can vary from 0-54% depending on various factors such as patient population, indication for renal biopsy, immunosuppressive regimen, and histological assessment [10-18]. The recurrence is 18-30% when indication biopsies are examined with immunofluorescence (IF) and electron microscopy (EM) in addition to light microscopy (LM) [10, 12]. The recurrence rate is even higher, that is, 43-54%, when protocol biopsies are performed and examined with the same histological methods (11, 16). Other major studies have reported 0-11.3% recurrence rates [13, 14, 17,19] with various immunosuppressive protocols. The posttransplant immunosuppressive regimen used in the studies predominantly included cyclosporine and azathioprine [10-17]. However, the posttransplant immunosuppressive regimen has changed over the years [14, 20]. In 1998, tacrolimus replaced cyclosporine, and by 2009, its use was universal [14, 20, 21]. Since 1996, mycophenolate mofetil (MMF) has been used more often than azathioprine [14, 20]. It is unknown whether changes in the posttransplant immunosuppressive regimen in recent decades affect the recurrence of LN.

We aimed to look at the recurrence rate of lupus nephritis and the outcome in the modern immunosuppressive era using a combination of MMF and tacrolimus. We hypothesized that this combination therapy would result in lower recurrence rates than historically reported

## Materials and methods

Patients

We identified our patients with a prior diagnosis of LN who had renal transplantation at James D. Eason Transplant Institute at Methodist University Hospital in Memphis, Tennessee, USA over 10 years (2006-2017) from our transplant database. Methodist University Hospital is a tertiary medical center and a major teaching affiliate of the University of Tennessee Health Science Center, Memphis, Tennessee, USA. These patients had a prior confirmed diagnosis of SLE and a biopsy-proven diagnosis of LN. The University of Tennessee Health Science Center, Memphis, Tennessee, institutional review board approved the study (IRB # 17-05386-XP).

Data collection

We conducted a retrospective chart review of the electronic medical records of all 38 identified patients from our database. We also reviewed their records in the United Network for Organ Sharing Network (UNOS) retrospectively. We collected demographic information (age, sex, race), body mass index (BMI), comorbidity information, tobacco use, alcohol use, age at onset of disease, follow-up period, type of donor (living versus deceased), type of dialysis, posttransplant biopsy, recurrence, graft loss, and death.

Renal biopsy

Patients underwent indication biopsy but not protocol biopsies. Biopsies were performed at the discretion of the transplant nephrologists when subjects developed abnormal urinalysis or decline in glomerular filtration rate (GFR) suggestive of a relapse of lupus nephritis.

Immunosuppression

The patients received standard treatment for posttransplant rejection, which consisted of induction therapy with thymoglobulin 4.5-6 mg/kg total over five days along with IV methylprednisolone: Day 0: 500 mg, followed by 250 mg day 1, 100 mg on day 2, 50 mg on day 3. This was followed by oral prednisone 20 mg/day to be tapered by 5 mg each week down to a maintenance dose of 5 mg/day. Concurrently, they were treated with MMF 500 mg by mouth twice daily starting on day 1. By day 4, the dose was increased to 1000 mg twice daily. Tacrolimus was initiated to maintain the trough level between 8-10 ng/ml in the first three months, followed by 6-8 ng/ml until one year, and then 6 ng/ml after that. Relapses of lupus nephritis in grafts were treated by prednisone burst and adjusting the MMF and tacrolimus doses.

Exposure

The primary exposure variable was the recurrence of LN. The recurrence was defined in two ways: biopsy-proven recurrence or recurrence based on laboratory results, e.g., persistent recurrent hematuria.

Outcomes

The primary outcome was the event of renal allograft loss or patient death, whichever happened first.

Statistical analysis

Descriptive data are summarized as categorical variables and mean± standard deviation (SD) or median (interquartile range). The associations between LN recurrence and combined outcome (death with functioning graft and or graft loss) were assessed using Cox proportional regression analysis. We also used the Kaplan-Meier method along with the Log-Rank test to assess the associations between LN recurrence and combined outcome (death with functioning graft and or graft loss). Proportional hazards assumptions were tested using scaled Schoenfeld residuals. As appropriate, results are presented as Hazard Ratio (HR) with 95% Confidence Intervals (95%CI).

## Results

Characteristics of kidney recipients with LN

The baseline characteristics of the 38 patients are summarized in Table 1.

**Table 1:** Baseline characteristics of kidney transplant recipients according to their LN recurrence status CAD: Cadaveric donor, LRD: Living related donor, HD: Hemodialysis, PD: Peritoneal dialysis, IQR: Interquartile range, LN: lupus nephritis *death after graft loss has been counted

	All Patients (n=38)	Recurrence (n=4)	No Recurrence (n=34)
Sociodemographic characteristics			
Age; (years) mean± SD	42±13	36±10	42±13
Gender; (female); n (%)	34 (89)	3 (75)	31 (91)
Race/Ethnicity; n (%)			
African American	34 (89)	4 (100)	30 (88)
Caucasian	3 (8)	0 (0)	3 (9)
Asian	1 (3)	0 (0)	1 (3)
Comorbidities:			
Body Mass Index; (kg/m2); mean± SD	26.8±5.3	28.2±4.9	26.6±5.4
Charlson Comorbidity Index; median (IQR)	3 (3-4)	3 (3-4)	3 (3-4)
Tobacco use; n (%)	6 (16)	1 (25)	5 (15)
Alcohol intake; n (%)	6 (16)	1 (25)	5 (15)
Transplantation related data:			
Age at onset of disease; (years) mean± SD	27±12	31±7	26±13
Follow-up; (days) median (IQR)	1,230 (460-2,227)	640 (335-2,212)	1,407 (460-2,227)
Type of Donor; (living) n (%)	9 (31)	1 (33)	8 (31)
Dialysis Duration; (years) median (IQR)	4 (2-8)	4 (3-5)	4 (2-8)
Type of dialysis; HD/PD/unknown	28/5/2	2/1/0	26/4/2
Death; n (%) *	3 (8)	1 (25)	2 (6)
Graft Loss; n (%)	9 (24)	1 (25)	8 (24)
	All Patients (n=38)	Recurrence (n=4)	No Recurrence (n=34)
Sociodemographic characteristics			
Age; (years) mean± SD	42±13	36±10	42±13
Gender; (female); n (%)	34 (89)	3 (75)	31 (91)
Race/Ethnicity; n (%)			
African American	34 (89)	4 (100)	30 (88)
Caucasian	3 (8)	0 (0)	3 (9)
Asian	1 (3)	0 (0)	1 (3)
Comorbidities:			
Body Mass Index; (kg/m2); mean± SD	26.8±5.3	28.2±4.9	26.6±5.4
Charlson Comorbidity Index; median (IQR)	3 (3-4)	3 (3-4)	3 (3-4)
Tobacco use; n (%)	6 (16)	1 (25)	5 (15)
Alcohol intake; n (%)	6 (16)	1 (25)	5 (15)
Transplantation related data:			
Age at onset of disease; (years) mean± SD	27±12	31±7	26±13
Follow-up; (days) median (IQR)	1,230 (460-2,227)	640 (335-2,212)	1,407 (460-2,227)
Type of Donor; (living) n (%)	9 (31)	1 (33)	8 (31)
Dialysis Duration; (years) median (IQR)	4 (2-8)	4 (3-5)	4 (2-8)
Type of dialysis; HD/PD/unknown	28/5/2	2/1/0	26/4/2
Death; n (%) *	3 (8)	1 (25)	2 (6)
Graft Loss; n (%)	9 (24)	1 (25)	8 (24)

The average age of our patient population was 42 years at the time of the renal transplant. The majority (89%) of patients were female and African Americans. The average duration of follow-up post-transplant was 3.4 years. The average age at the time of diagnosis of SLE was 27 years old. Most patients received hemodialysis before transplant for a median duration of four years. Most of them received cadaveric donors for renal transplantation.

Relapse rate

**Figure 1:**
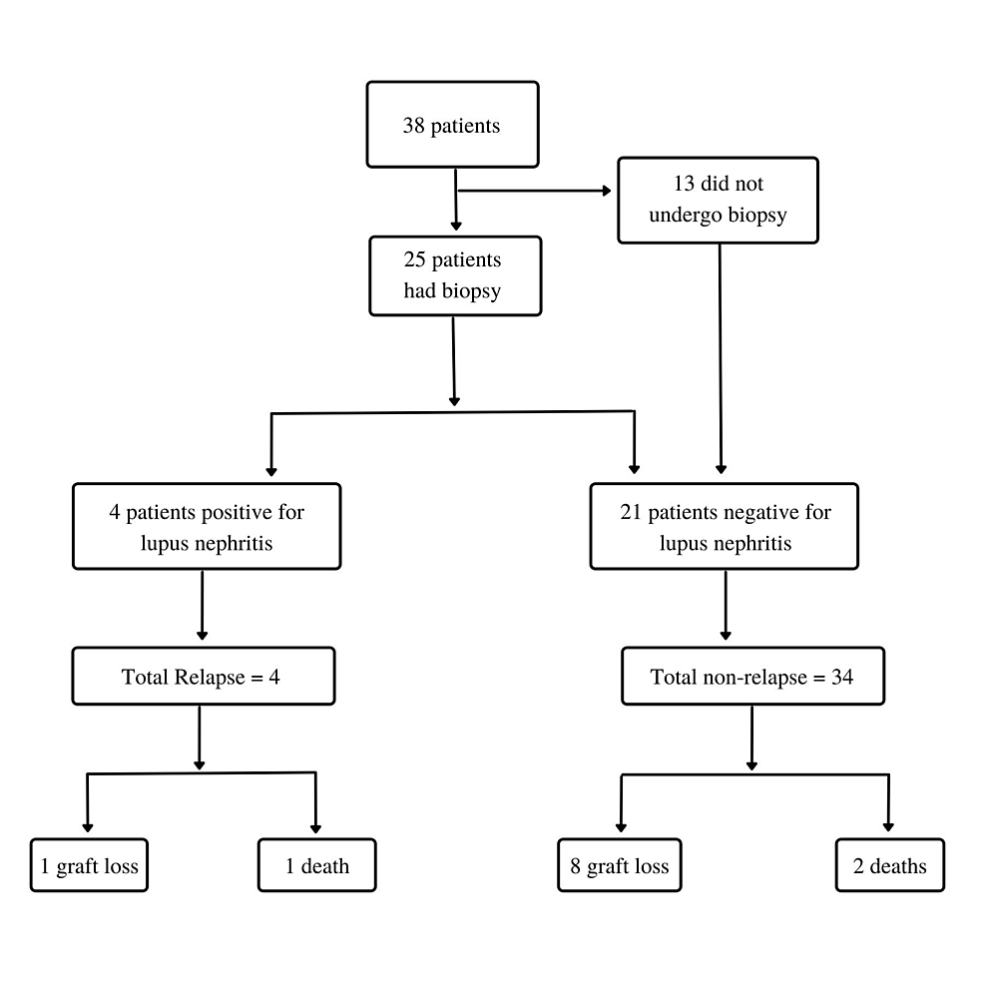
Flow chart of patient selection

Figure 1 shows the patient selection. Out of the 38 subjects, 25 underwent post-transplant biopsy. Four of the 38 patients (10.5%) among the total patients (n=38) had a recurrence of lupus nephritis. Among the 25 patients who underwent biopsy, four had a relapse of lupus nephritis (16%).

Graft loss and survival

**Figure 2:**
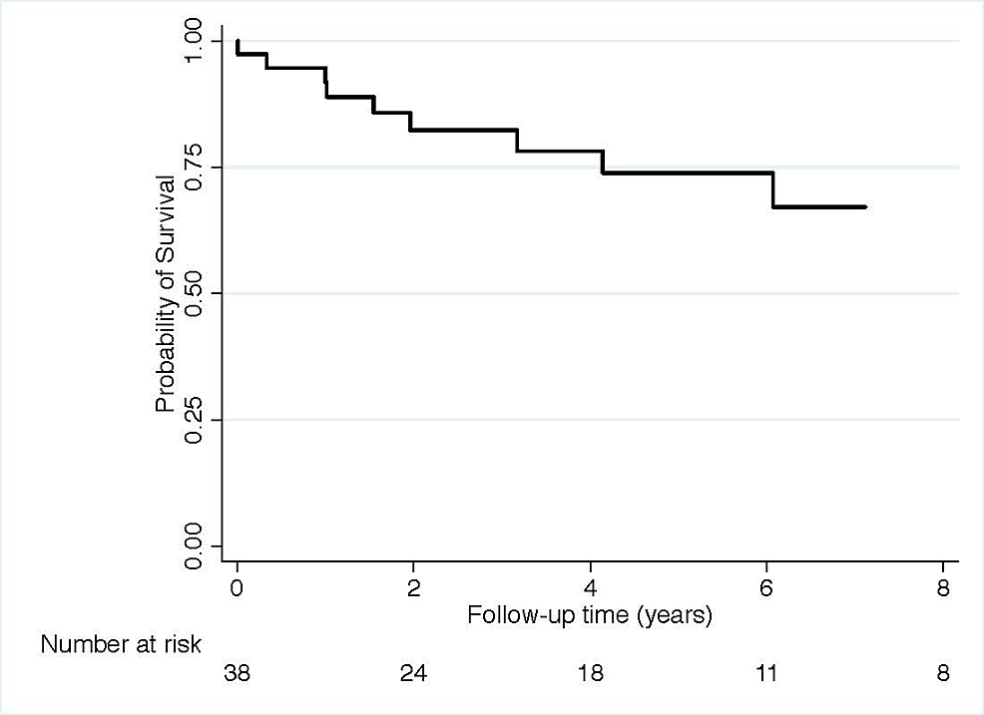
Probability of death with functioning allograft or graft loss in the entire cohort

A total of 10 patients (26%) had graft loss (n=9) or death (n=3, including two patients with previous graft loss) during the median follow-up time of 1230 days (IQR: 460-2227 days). Table 1 shows that three patients died and nine patients lost their functioning graft during this follow-up period. Figure 2 depicts the overall survival of patients; 75% of patients had a functioning allograft five years after transplantation.

**Figure 3:**
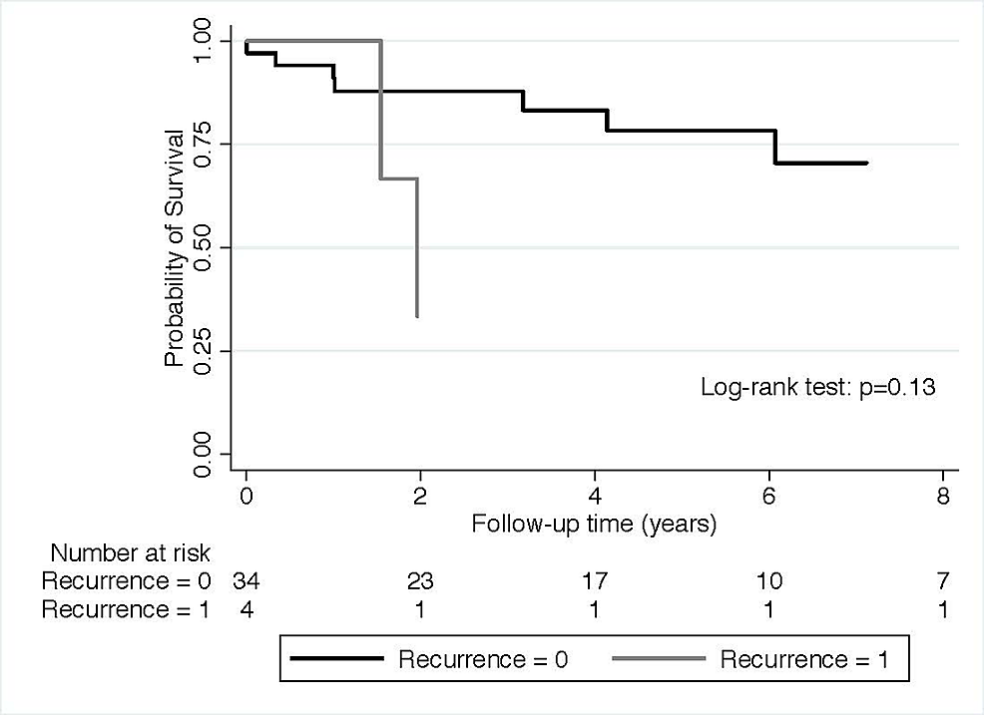
Probability of death with functioning allograft or graft loss in patients with biochemical/biopsy evidence of recurrence of lupus nephritis versus those without recurrence

**Figure 4:**
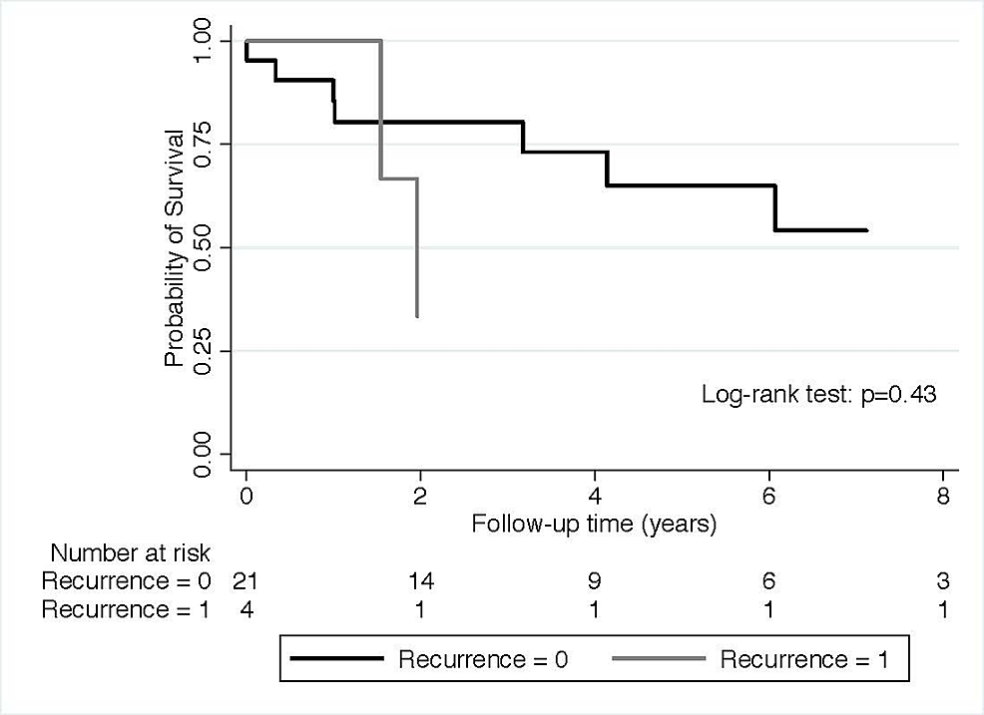
Probability of death with functioning allograft or graft loss in patients with biopsy-proven recurrence of lupus nephritis versus those without recurrence

Figure 3 shows that recipients with biochemical evidence of recurrence had an overall trend toward worse combined graft loss and death probability than those without any sign of recurrence of LN. Qualitative results were found in a cohort of patients with biopsy-proven recurrence (Figure 4).

Recipients with biopsy-proven recurrence had an overall trend toward worse combined graft loss and death probability than those without any sign of recurrence of LN. In addition, patients with biochemical or biopsy-proven LN recurrence showed a trend for increased risk for graft loss or death (Hazard Ratio= 3.14, 95%Confidence Interval: 0.65-15.24) compared to the recipient without recurrence in our unadjusted proportional Cox regression model. Similarly, patients with biopsy-proven LN recurrence showed a trend for increased risk for graft loss or death (Hazard Ratio= 1.86, 95%Confidence Interval: 0.38-9.03) compared to the recipient without recurrence in our unadjusted proportional Cox regression model.

## Discussion

In our study, the LN recurrence rate was 11% among the 38 patients who received a renal transplant for ESRD secondary to lupus nephritis over 10 years (2006-2017). A majority (65%) of our patients underwent a biopsy for abnormal urinalysis or abnormal renal function at the discretion of the treating nephrologist. The recurrence rate was 16% among the patients who underwent indication biopsy (n=25).

Recurrence of LN in the allograft can present with worsening graft function, new-onset proteinuria, and or new-onset hematuria [8]. The median time to recurrence was 4.3 years but can vary from five days post-transplant to 16 years [9, 10, 12, 19, 20]. However, recurrent lupus nephritis differs from incidental histologic findings on the allograft without clinical renal findings such as a change in graft function, hematuria, proteinuria, or other renal manifestations of lupus [8]. Recurrent lupus nephritis was present in 2-11% (8). Recurrence of SLE in the allograft occurs in 0-54% among lupus nephritis patients who have undergone renal transplants [4, 8, 22, 23]. The wide variation in relapse rate is due to various factors [10, 12, 13, 19, 21]. The period prevalence of recurrent lupus nephritis was 2.44% [14]. The data reported from the UNOS database could have underestimated the relapse as there was no histologic confirmation (19). Using both immunofluorescence (IF) and electron microscopy (EM), in addition to light microscopy (LM), investigators found a higher recurrence rate, i.e., 18-30%, compared to other studies with indication biopsy alone [10, 12]. In a study of 50 patients, Goral et al. reported 50% relapse in patients who underwent biopsy and 30% of total patients [12]. In addition, other studies using protocol biopsies reported a higher recurrence rate of up to 50% [16, 19]. Our study reported a slightly lower relapse rate compared to other studies, where indication biopsy was done. Three out of four patients who relapsed were African Americans, female, and an average age of 36 years. Our numbers are too small to identify any single factor to account for the lower relapse rate. In general single-center studies have reported a higher recurrence rate because those studies are done using indication biopsies [24-27].

Overall, the low relapse rate in renal transplant patients might be attributed to the immunosuppressive regimen, burned-out effect of uremia, and dialysis [24-27]. Non-Hispanic Black race, female gender, and age <33 years independently were associated with increased risk of recurrence (14). Markers of disease activity such as elevated anti-dsDNA antibodies and low complement levels are reported to be unreliable predictors of relapse [4]

The risk of graft failure is increased secondary to relapse of lupus nephritis, but actual graft loss is rare, as reported mainly through studies from a single center [10, 12-14, 28]. Our results are like previous findings from earlier studies. Out of the nine graft losses reported in our study, only one was due to recurrence of lupus. Three total deaths were reported, and only one was due to recurrence of lupus. In our study, recurrence was associated with decreased survival compared to previous studies. This could be due to the small sample size in our case. In earlier studies, recurrence of LN in allograft transplants had no effect on patient survival as the relapse of lupus nephritis in the allograft was minimal [13, 29]. The relapsed lesions were mostly WHO CLASS I & II like the findings reported by other investigators [12, 16, 18]. The clinical significance of this subclinical recurrence is not clear because there is no longitudinal biopsy follow-up in patients with relapsed subjects. The authors suggested that introducing a newer posttransplant immunosuppressive regimen might have prevented the development of proliferative lesions in the graft and recurrent lupus nephritis [13]. In contrast, clinical recurrent lupus nephritis can cause deterioration of renal function [13, 30]. Rejection and chronic allograft nephropathy account for the significant cause of allograft loss in transplanted kidneys [14]. It is likely that protocol biopsies with IF and EM would likely show more relapses but may not affect ultimate graft loss and patient survival. For this reason, the effect of recurrence of lupus on graft outcome is minor [29]. 

The effect of immunosuppression on the recurrence of lupus nephritis in the graft kidney varies among studies. The recurrence rate was higher (3.9% vs. 1.98%) among patients who were transplanted before January 1996 versus patients transplanted after January 1996 [14]. Azathioprine was associated with a higher rate of recurrence (odds ratio of 1.38) but was not statistically significant [14]. Azathioprine was found to have a protective effect on relapse of lupus nephritis, but both tacrolimus and MMF have unfavorable effects on relapse [13]. MMF was associated with better graft and patient survival [13]. Azathioprine and tacrolimus do not affect survival and graft failure [13]. The authors conceded that they did not adjust other confounders such as dose and duration of treatment. Similar effects of MMF have been reported in another study [31]. There was no significant difference in maintenance immunosuppression between relapse and non-relapse lupus nephritis patients [10, 16]. Çeltİk et al. suggested that treatment with anti-thymocyte globulin (ATG) and MMF have a protective effect on recurrence [19]. Our patients are treated similarly with MMF and tacrolimus, and the potential effects of any agent cannot be verified from the current study. Other studies conducted in the comparable period to our study were treated with similar immunosuppressive agents are summarized in Table 2.

**Table 2:** Published studies between 2016-2020 for assessing recurrence of lupus nephritis ATG: Antithymocyte globulin, AZA: Azathioprine, CS: Corticosteroids, CsA: Cyclosporine, MMF: Mycophenolate mofetil, TAC: Tacrolimus, USRDS: United States Renal Data System, UNOS: United Network for Organ Sharing

place	Time period	Year of publication	Number of patients	Follow-up year median	Incidence of recurrence	Post-transplant treatment regimen
Gdansk, Poland[32]	1999-2014	2016	19	0.1-10.5	1 (5%)	CS, Csa, TAC, ATG, MMF, Basiliximab
Medellín, Colombia [33]	2005–2013	2016	27	N/A	1 (4%)	Alemtuzumab, dacilizumab, ATG, Basiliximab, Csa, MMF, AZA, TAC, CS
Izmir, Turkey[19]	2000–2013	2016	12	4.8 (1.1-10.6)	50%	CS, Csa, MMF, AZA, TAC, ATG, Basiliximab
ANZDATA registry, Australia and New Zealand	1998–2012	2016	176	3.8 [1.6–8.3]	4 (2%)	N/A
Cali, Colombia^ [34]^	1996-2014	2017	65	7.2 [3.2–11.7	2 (3%)	CS, AZA, CsA, MMF, Everolimus, Sirolimus
USRDS/UNOS, US[22]	1996–2011	2017	5884	4.7 [2.0–8.3]	67 (1.1%)	Alemtuzumab, daclizumab, ATG, Basiliximab, CsA, MMF, AZA, TAC, ATG, CS, Sirolimus
Seoul, Republic of Korea[34]	2005–2016	2018	19	5.8 ± 2.7	0 (0%)	CS, TAC, MMF
Seoul, Republic of Korea[35]	1998-2017	2020	28	9.5	N/A	ATG, Basiliximab, CS, CsA, TAC, MMF

Our study has several limitations. First and foremost, our study is retrospective in nature. Secondly, our subjects had an indication biopsy, which may not reflect the true relapse as indicated by other studies where protocol biopsies are done. Additionally, we only had a follow-up median of 3.7 years. Other studies have indicated that relapse can occur up to 16 years.

## Conclusions

Compared to earlier studies, we have reported lower recurrence of lupus nephritis among subjects treated with a posttransplant treatment regimen using MMF and tacrolimus. Therefore, it is possible that the newer regimen may have minimized the recurrence rate but needs further confirmation.
